# Medical Society of Tennessee

**Published:** 1845-07

**Authors:** 


### MEDICAL SOCIETY OF TENNESSEE.

We are indebted to the politeness of our friends for two copies of the Proceedings of the Medical Society of Tennessee. From the published report we gather a few items not communicated to us in the letter, the substance of which we gave in our last number. We copy the most interesting.

Dr. Manlove read the history of "a case of Gastrotomy, which was resorted to for the relief of obstruction in the bowels, terminating in Artificial Anus, which eventually healed without a surgical operation. He also presented the patient for the examination of the members of the Society." This case will appear in the Boston Medical and Surgical Journal, from which we shall take pleasure in copying it.

Dr. Irwin reported a case of "lodgment of a river pebble in the bowels, attended with very severe symptoms, which subsided by its discharge through the natural passage."

The Mesmeric committee, appointed at the last meeting of the Society, were not able to make a satisfactory report, and asked for time to continue their investigations. It appears that the great hindrance was "the *scarcity of patients*, and the *difficulty of procuring them*." It is insuperable, we apprehend.

The Museum, commenced a few years ago in connection with the Society, does not grow as it ought. Members are negligent about sending specimens of morbid anatomy. Few preparations, according to the committee, were received during the last year.

Again, as we have so often done, we wish this Society long life and abundant prosperity. Y.

